# Predicting grain protein content of field-grown winter wheat with satellite images and partial least square algorithm

**DOI:** 10.1371/journal.pone.0228500

**Published:** 2020-03-11

**Authors:** Changwei Tan, Xinxing Zhou, Pengpeng Zhang, Zhixiang Wang, Dunliang Wang, Wenshan Guo, Fei Yun

**Affiliations:** 1 Jiangsu Key Laboratory of Crop Genetics and Physiology/Jiangsu Co-Innovation Center for Modern Production Technology of Grain Crops/Joint International Research Laboratory of Agriculture and Agri-Product Safety of the Ministry of Education of China, Yangzhou University, Yangzhou, China; 2 National Tobacco Cultivation and Physiology and Biochemistry Research Centre/Key Laboratory for Tobacco Cultivation of Tobacco Industry, Henan Agricultural University, Zhengzhou, China; Universidade Federal de Uberlandia, BRAZIL

## Abstract

Remote sensing has been used as an important means of modern crop production monitoring, especially for wheat quality prediction in the middle and late growth period. In order to further improve the accuracy of estimating grain protein content (GPC) through remote sensing, this study analyzed the quantitative relationship between 14 remote sensing variables obtained from images of environment and disaster monitoring and forecasting small satellite constellation system equipped with wide-band CCD sensors (abbreviated as HJ-CCD) and field-grown winter wheat GPC. The 14 remote sensing variables were normalized difference vegetation index (NDVI), soil-adjusted vegetation index (SAVI), optimized soil-adjusted vegetation index (OSAVI), nitrogen reflectance index (NRI), green normalized difference vegetation index (GNDVI), structure intensive pigment index (SIPI), plant senescence reflectance index (PSRI), enhanced vegetation index (EVI), difference vegetation index (DVI), ratio vegetation index (RVI), R_blue_ (reflectance at blue band), R_green_ (reflectance at green band), R_red_ (reflectance at red band) and R_nir_ (reflectance at near infrared band). The partial least square (PLS) algorithm was used to construct and validate the multivariate remote sensing model of predicting wheat GPC. The research showed a close relationship between wheat GPC and 12 remote sensing variables other than R_blue_ and R_green_ of the spectral reflectance bands. Among them, except PSRI and R_blue_, R_green_ and R_red_, other remote sensing vegetation indexes had significant multiple correlations. The optimal principal components of PLS model used to predict wheat GPC were: NDVI, SIPI, PSRI and EVI. All these were sensitive variables to predict wheat GPC. Through modeling set and verification set evaluation, GPC prediction models' coefficients of determination (R^2^) were 0.84 and 0.8, respectively. The root mean square errors (RMSE) were 0.43% and 0.54%, respectively. It indicated that the PLS algorithm model predicted wheat GPC better than models for linear regression (LR) and principal components analysis (PCA) algorithms. The PLS algorithm model’s prediction accuracies were above 90%. The improvement was by more than 20% than the model for LR algorithm and more than 15% higher than the model for PCA algorithm. The results could provide an effective way to improve the accuracy of remotely predicting winter wheat GPC through satellite images, and was conducive to large-area application and promotion.

## Introduction

The grain quality index of winter wheat includes many parameters, of which grain protein content (GPC) has been the most important indicator for measuring wheat quality [1–3]. At present, GPC was mainly detected by chemical determination method based on manual measurement, which was costly and inefficient. In addition, the current sampling method for investigating the quality of winter wheat was point-like sampling. It meant that only a few sampling points were used to reflect the situation in a large area. Therefore, the samples lacked representativeness and made it difficult to grasp the overall quality information of winter wheat over requisite time. In the protein content monitoring method, a combined method was proposed for pretreatment of the NIR spectrum. This was based on both the empirical mode decomposition and the wavelet soft-threshold methods, presuming certain accuracy in the monitoring of GPC content [4]. A rapid and simplified decision support method to predict the wheat quality at a small range was established with an accuracy of more than 80% [5]. Compared with previous researches, remote sensing technology has the advantages of being fast, accurate and based on wide range in data collection. Therefore, the model based on remote sensing and corresponding algorithm could serve as an effective way to obtain wheat quality status in advance. With the urgent need for remote sensing in the agricultural field, more and more studies focused on crop quality prediction. To materialize this, the remote sensing has a wide range of application and development in large-scale regional crop management and monitoring [6, 7].

For many years, agricultural remote sensing focused mainly on crop growth monitoring and yield estimation, and formed a relatively complete technical system. Recently, by using different combinations of remote sensing variables, the reliability of remote sensing model of nitrogen concentration in wheat leaves has been improved [8]. Likewise, the remote sensing prediction of crop yield loss under soil salinization effect has also achieved some results [9]. Production forecasts based on advance very high-resolution radiometer (AVHRR) data in Kansas, USA were almost identical to production data from local government field surveys [10]. At present using multi-temporal radarsat-2 SAR image, wheat could be identified effectively with an accuracy rate of 0.929 [11]. The normalized difference vegetation index (NDVI), which extracted from the moderate resolution imaging spectroradiometer (MODIS) data, has been used in a wide range of applications for global agricultural monitoring, particularly in crop growth monitoring, quality prediction and yield estimation [12]. In another attempt from 2003 to 2015, using NDVI deduced MODIS data some researchers improved the estimation and prediction method of wheat yield in Hungary with good results [13]. However, there were few reports on crop quality remote sensing prediction using spectral reflectance. Later on, through the ground spectral data, some studies have assessed the metabolic energy, ash content, crude protein and other indicators of leguminous plants [14]. There were also corresponding breakthroughs in forestry, as well as the water index of olive forest was successfully detected by vegetation spectroscopy [15]. In recent years, many researchers studied the prediction of crop quality based on the space satellite remote sensing platform [16, 17]. With advancement of geospatial technology in agriculture and the significant improvement of the resolution of remote sensing images, a large number of studies on the spatial pattern of farmland yield and quality have been reported successively [18]. Early repeated remotely sensed multispectral data reliably predicted the yield and quality of winter wheat and spring barley [19]. In the monitoring of quality fluctuation, some researchers combined remote sensing with geographic information system (GIS) to explain the changes of soybean oil and protein content [20]. Multi-temporal image monitoring might be the future trend. Recent study has also shown that three satellite images from each of landsat thematic map (TM) and advanced synthetic aperture radar (ASAR) successfully monitored the crop conditions and predicted yield and protein content [21]. According to a number of previous studies, remote sensing technology has been considered as a potential and effective method to predict the protein content and quality of wheat grains [22]. The results showed that the prediction of wheat GPC with TM and enhanced thematic mapper (ETM) data was effective [23]. Studies also suggest feasibility of using KODA-CIR (Eastman Kodak Co., USA) and Cropscan (NextInstruments Co., Australia) data to predict GPC of winter wheat one month before the harvest [24]. GPC prediction by using high-resolution satellite images to monitor the potential growth and development of wheat was also available [25]. Besides, the fusion of multi-sensor and multi-temporal remote sensing images as the data source provided a technical approach for predicting wheat GPC [26]. There had been many reports on remote sensing monitoring of agricultural conditions based on partial least squares method. Most of these primarily focused on crop pests and diseases as well as growth. Some researchers had successfully measured the canopy biomass and nitrogen status of wheat by using NDVI and partial least square (PLS) algorithm. In the growth of rice leaves, there were a number of breakthroughs in hyperspectral reflectance and PLS regression analysis [27–28]. Based on multi-temporal and multi-season satellite remote sensing data, PLS algorithm was used to monitor the host species distribution of spruce budworm in large forests [29]. However, there were very limited reports on quantitatively forecasting chemical components in grains such as GPC using satellite remote sensing data [8, 30]. On September 6, 2008, China has launched successfully satellites A and B (abbreviated as HJ-CCD) of the “Environment and Disaster Monitoring and Forecasting Small Satellite Constellation System” with independent intellectual property rights. The satellites were equipped with wide-band CCD sensors with spatial resolution of the sensor being 30 m. Time resolution was 2 d when satellites A and B were making observations simultaneously. This made them an ideal data source for agricultural remote sensing operation. Some studies on remote sensing prediction of wheat quality were still based on traditional algorithms and its accuracy was consequently affected [17, 20]. In this study, HJ-CCD images were used as remote sensing data sources and combined with PLS algorithm to construct GPC prediction model.

The objectives of the present study were to investigate the quantitative relationship between satellite remote sensing variables during flowering period and wheat GPC, and developed an effective way to improve the accuracy of predicting wheat GPC through remote sensing.

## Materials and methods

### Test design and data acquisition

Field sampling was used in this study for three years. The survey area was representative and the varieties are different. Samples were taken back to the laboratory for analysis, and corresponding satellite image data were collected.

For the present investigation, data collection was carried out in 5 counties namely, Taixing, Jiangyan, Yizheng, Xinghua and Dafeng in Jiangsu Province, the Peoples Republic of China. There were 15–20 sampling points in each county, totaling 92. The location of each sampling site was determined by using a Juno ST hand-held GPS meter (Trimble Co., USA). The survey mainly included information collection on winter wheat varieties, growth period, population growth and disaster status (mainly by pests and diseases). Winter wheat varieties were of medium and weak gluten type, mainly *Yangmai 13*, *Yangmai 15 and Yangmai 16*. These varieties were available in the experimental counties. At harvest time, wheat grains were sampled by five-point sampling method in the field, and then brought back to the laboratory for wheat GPC determination [31].

A total of 3 tests were launched in the experimental counties from 2016–2018 to collect data. The satellite data was HJ-CCD images taken at flowering stage of the wheat crop. Data collection for Test 1, 2 and 3 were conducted on May 2, 2016; April 24, 2017 and April 26, 2018, respectively. The sampling points considered for the Test 1–3 were 92, 96 and 67, respectively.

### Image preprocessing

Environment for Visualizing Images (ENVI 5.4) software (ESRI Co., USA) was used to preprocess satellite images. Firstly, the geometric rough correction of the satellite image was carried out by using the 1:100,000 topographic maps of Jiangsu area. Thereafter, the GPS control points for ground measuring were used to precisely correct the satellite image. This helped to ensure that the precision of geometric correction was better than one pixel. Atmospheric correction and reflectance conversion were carried out by empirical linear method [32]. According to the analysis of the results, the corresponding single-band value graph was obtained by band math. Data of wheat growing areas were obtained by supervised classification. The winter wheat planting data were superimposed and the non-winter wheat area was eliminated by one-to-one solution and binarization mask. By using the administrative boundary vector data and the PLS model, the spatial distribution map of winter wheat GPC prediction in Jiangsu province was produced.

### Satellite remote sensing variables

In combination with the physical significance of spectral indices, selection of model parameters was based on the spectral characteristics of crops and the available literatures at home and abroad. Finally, in this study, four HJ-CCD bands and ten common spectral vegetation indices were selected (Table 1) as independent variables for PLS analysis in order to construct the model of predicting winter wheat GPC.

**Table 1:** Remote sensing vegetation indices used in this study.

Vegetation index	Abbreviation	Algorithm	Source
Normalized difference vegetation index	NDVI	(R_nir_-R_red_)/(R_nir_+R_red_)	[33]
Soil-adjusted vegetation index	SAVI	(R_nir_-R_red_)/(R_nir_+R_red_+0.5)*1.5	[34]
Optimized soil-adjusted vegetation index	OSAVI	(R_nir_-R_red_)/(R_nir_+R_red_+0.16)*1.16	[35]
Nitrogen reflectance index	NRI	(R_green_-R_red_)/(R_green_+R_red_)	[36]
Green normalized difference vegetation index	GNDVI	(R_nir_-R_green_)/(R_nir_+R_green_)	[37]
Structure intensive pigment index	SIPI	(R_nir_-R_blue_)/(R_nir_+R_blue_)	[38]
Plant senescence reflectance index	PSRI	(R_red_-R_blue_)/B_nir_	[39]
Enhanced vegetation index	EVI	2.5*(R_nir_-R_red_)/(R_nir_+6*R_red_-7.5*R_green_+1)	[40]
Difference vegetation index	DVI	R_nir_-R_red_	[41]
Ratio vegetation index	RVI	R_nir_/R_red_	[42]

R_blue_, R_green_, R_red_ and R_nir_ denoted spectral reflectance at blue, green, red and near infrared bands, respectively.

To extract spectral reflectance values of corresponding GPS positioning sampling points, ENVI 5.4 and geographic information system software (ArcGIS 10.2) (ESRI Co., USA) were used. These combined with the remote sensing vegetation index algorithm as provided in Table 1, satellite remote sensing variables were calculated using Excel 2016.

### PLS regression

PLS regression was first applied to the field of chemometrics. Since then, it has been considered as a new multivariate analysis method with wide applicability. It was concentrated on the characteristics of principal component, linear regression and typical multiple regression analyses. It could effectively solve many problems. Such as, problems that cannot be solved by ordinary multiple regression, especially when there were many variables and multiple correlations. In these cases, PLS could effectively decompose and screen the comprehensive variables that were most explanatory to the dependent variables. Therefore, the established model is more reliable than the ordinary regression analysis. The PLS method first extracted a new variable called component as an independent variable, and established a linear combination relationship between the dependent variable and the independent variable. The coefficient was determined by PLS calculation, and then the regression equation of the dependent variable was constructed. The regression model established by the PLS method could be expressed by Eq 1:

$$y_{m}=a_{0m}+a_{1m}x_{1}+\cdots +a_{Pm}x_{p}(m=1,2,\ldots p)$$ 1

Where *x*_1_,⋯*x_p_* were linear combinations of remote sensing variables, *a*_0*m*_,*a*_1*m*_,⋯*a_pm_* were parameters of the regression model and could be computed by PLS.

When the model was established by PLS algorithm, the increase of the number of principal components would improve the accuracy of the model. But too many principal components would cause over-fitting and the error would increase. Therefore, it was very important to determine the optimal principal components number of the PLS model. In this study, the sum of squared residuals was calculated by the cross-validation method. The smaller the predictive residual errors sum of square (PRESS) value, the stronger the prediction ability of the model is. Therefore, the optimal principal components number could be determined according to the minimum value of PRESS. PRESS can be expressed by Eq 2:

$$PRESS=\sum_{i=1}^{k}{\left(y_{i}-y_{i,-i}\right)}^{2}$$ (2)

Where *y_i_,y_i,−i_* were the measured value corresponding to the *i*th sample and the estimated value when the *i*th sample was excluded, and *k* was the number of validating iterations.

For the basic principles and specific practices of the PLS algorithm and PRESS, please refer to reference [43], which is not described here. Both the PLS and PRESS processes were performed by a self-written MATLAB program.

### Evaluation of the model

Using the samples of the modeling set, and the verification set, the model was evaluated by plotting the 1:1 relationship graph between the predicted and measured values of winter wheat GPC. The evaluation indices were the determination coefficient (R^2^) and the root mean square error (RMSE) [44]. On one hand, the larger the R^2^, the better the model is. On the other hand, the smaller the RMSE, the stronger the estimation ability of the model is. RMSE and estimation accuracy were calculated using Eqs 3 and 4, respectively:

$$RMSE=\sqrt{\frac{1}{n}\sum_{i=1}^{n}{\left(y_{i}-{\hat{y}}_{i}\right)}^{2}}$$ (3)

$$Accuracy=\frac{1}{n}\sum_{i=1}^{n}\left|y_{i}-{\hat{y}}_{i}\right|$$ (4)

Where *y*_*i*_ and ${\hat{y}}_{i}$ represented measured values and predicted values of winter wheat GPC, respectively, and *n* was the number of samples.

## Results

### GPC distribution

The GPC data measured in Tests 1–3 were arranged in the order of the GPC values in the winter wheat grain sample. To enhance the stability of the prediction model, under the premise that the maximum and minimum values of winter wheat GPC were guaranteed, needs to be included in the modeling sample set. To perform this, the numerical samples of 255 GPC were randomly divided into modeling set and verification set according to the ratio of 3:2. It could be seen from Table 2 that the amplitude of variation, mean, standard deviation and standard error of the modeling set and verification set samples were similar. At the same time, the modeling set and the verification set samples had desirable consistency.

**Table 2:** Distribution of winter wheat GPC in the modeling and verification set (GPC unit: %).

Sample set	Number of samples	Amplitude of variation	Mean	Standard deviation	Standard error
Modeling set	153	9.36–14.58	11.99	1.33	0.11
Verification set	102	9.38–14.39	12.29	1.42	0.14

GPC refered to the grain protein content in dry matter.

### Quantitative analysis between remote sensing variables and GPC

Table 3 shows the quantitative analysis of the GPC and remote sensing variables of 153 samples in the modeling set. It indicated that there was significant or extremely significant relationship between the GPC and 12 remote sensing variables except R_blue_ and R_green_. The GPC was most closely related to NDVI, followed by enhanced vegetation index (EVI). The correlation coefficients (*r*) being 0.82 and 0.75, respectively for NDVI and EVI. The correlation between vegetation index and GPC was obviously better than single-band. All the other remote sensing variables had considerable multiple pairwise correlations. Except PSRI and R_blue_, R_green_ and R_red_, other remote sensing variables had pairwise correlation coefficients between 0.80 and 0.99. In particular, single-band B1-B4 pair wise correlation coefficients were between 0.93 and 0.98, and the pairwise correlation coefficient of most vegetation indices were above 0.90.

**Table 3:** Correlation coefficients (r) between remote sensing variables and GPC.

	GPC	R_blue_	R_green_	R_red_	R_nir_	NDVI	OSAVI	SAVI	SIPI	PSRI	GNDVI	NRI	RVI	DVI	EVI
R_blue_	-0.22	1.00													
R_green_	-0.08	0.98	1.00												
R_red_	-0.46	0.97	0.96	1.00											
R_nir_	0.51	0.93	0.93	0.96	1.00										
NDVI	0.82	-0.67	-0.78	-0.88	0.93	1.00									
OSAVI	0.65	-0.67	-0.79	-0.85	0.94	0.95	1.00								
SAVI	0.59	-0.65	-0.81	-0.87	0.96	0.94	0.98	1.00							
SIPI	0.71	-0.64	-0.71	-0.69	0.95	0.98	0.97	0.98	1.00						
PSRI	0.63	-0.37	-0.26	-0.18	0.77	0.86	0.93	0.98	0.91	1.00					
GNDVI	0.67	-0.62	-0.79	-0.92	0.64	0.95	0.88	0.91	0.92	0.97	1.00				
NRI	-0.59	-0.68	0.68	0.87	-0.58	-0.87	-0.88	-0.86	-0.86	0.90	0.85	1.00			
RVI	0.61	-0.69	-0.82	-0.84	0.94	0.99	0.99	0.99	0.97	0.83	0.87	-0.84	1.00		
DVI	-0.63	0.66	0.72	0.77	-0.88	-0.97	-0.96	-0.96	-0.96	0.86	0.85	0.85	0.99	1.00	
EVI	0.75	-0.64	-0.78	-0.79	0.97	0.99	0.99	0.99	0.99	0.94	0.87	-0.83	0.98	0.98	1.00

All abbreviations were denoted by: normalized difference vegetation index (NDVI), soil-adjusted vegetation index (SAVI), optimized soil-adjusted vegetation index (OSAVI), nitrogen reflectance index (NRI), green normalized difference vegetation index (GNDVI), structure intensive pigment index (SIPI), plant senescence reflectance index (PSRI), enhanced vegetation index (EVI), difference vegetation index (DVI), ratio vegetation index (RVI), R_blue_ (reflectance at blue band), R_green_ (reflectance at green band), R_red_ (reflectance at red band) and R_nir_ (reflectance at near infrared band)

### Determination of the number of optimal principal components

**Fig 1:**
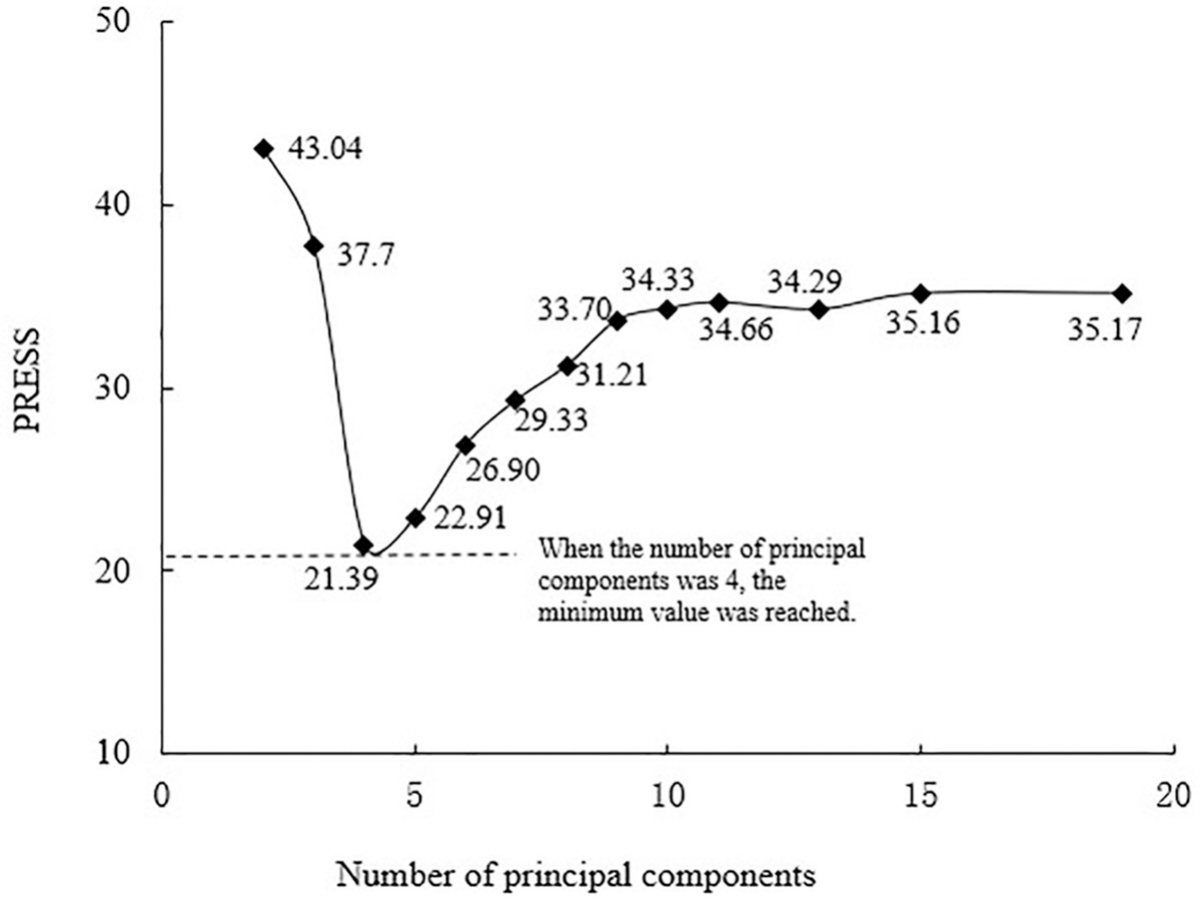
PRESS changes with the principal components.

The smaller the PRESS values, the stronger the prediction ability of the model is. It means the number of optimal principal components could be determined based on the PRESS minimum value. Fig 1 shows the variation of PRESS with the number of principal components obtained from the GPC modeling set. At the beginning, as the number of principal components increased, the PRESS value decreased to a large extent. It has indicated that due to the small number of principal components, the model fitting was extremely inadequate. It means the missing fitting phenomenon occurred. When the principal components number of the GPC model was 4, the PRESS value was the smallest (PRESS = 21.39). After that, as the number of principal components increased, the PRESS value increased sharply, until they tend to be saturated. Via this, it was indicated that the over-fitting phenomenon occurred due to too many principal components. Therefore, it was reasonable to select the number of principal components corresponding to the minimum PRESS value. Since the optimal principal components number of the PLS model, the optimal principal components number of the GPC model based on PLS algorithm were 4.

### PLS model

The structure of the PLS model was based on the PLS algorithm and the four vegetation indices with the principal components number of 4. All these were sensitive to the prediction of wheat GPC and could be easily extracted and calculated from the HJ-CCD image. These were NDVI, structure intensive pigment index (SIPI), plant senescence reflectance index (PSRI) and EVI. All these were considered as the independent variables and the GPC was the dependent variable for the PLS model of predicting the GPC. The GPC model constructed by the modeling set and the HJ-CCD images during the three days 2016-05-02, 2017-04-24, and 2018-04-26 were Eq 5:

GPC=3.873×NDVI+1.696×SIPI+2.862×PSRI‐1.276×EVI+5.821 (5)

**Fig 2:**
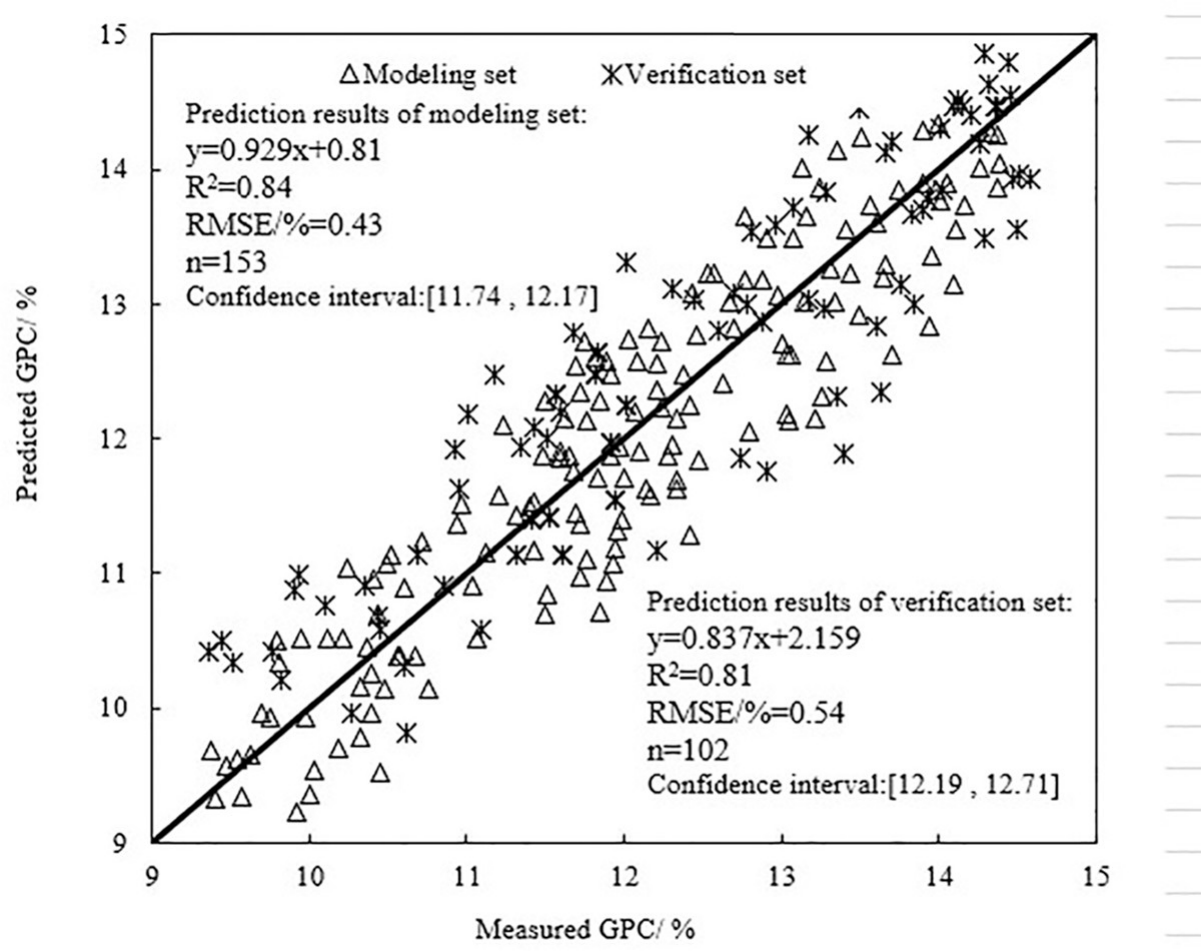
Evaluation of GPC model based on PLS algorithm.

After the PLS model was built, it was used to predict winter wheat GPC. The predicted and measured GPC values were plotted as a 1:1 scatter plot. The optimal linear regression equation and its R^2^ and RMSE were obtained. Fig 2 shows the evaluation of the PLS model's prediction ability. It could be seen from Fig 2 that the model set samples number was larger than the verification set samples number. The R^2^ of the linear equation thus established by the modeling set was larger than R^2^ of the verification set. The set RMSE was significantly smaller than the verification set RMSE. It indicates that the prediction model effect of the modeling set samples was better than the verification set. Thereby, it has theoretically conformed to the model's evaluation law [45]. In addition, the R^2^ values between the predicted and measured GPC of the modeling and verification sets were greater than 0.8 and the RMSE were 0.43% and 0.54%, respectively. This result indicated that the PLS model could be used effectively to predict the winter wheat GPC.

In order to compare with the traditional algorithm, the linear regression (LR) and principal components analysis (PCA) algorithm were used to establish the GPC estimation models using the model set and verification set samples, respectively. The GPC models were evaluated by R^2^ and RMSE. The specific process was not described here. Table 4 shows the comparison of predicted results with PLS, LR and PCA based on the modeling set and verification set. It showed that the sample number was the same. The PLS algorithm models’ R^2^ were greater than those for LR and PCA algorithm models. But RMSE were smaller than those for LR and PCA algorithm models. This indicated that the PLS algorithm model was better than the LR and PCA algorithms in predicting winter wheat GPC. The modeling set and the verification set prediction accuracy were 20.6% and 22.4% higher than the LR algorithm, respectively, and were 15.4% and 16.3% higher than PCA algorithms, respectively.

**Table 4:** Comparison of predicted abilities with PLS, LR and PCA.

Algorithm	Number of principal components	Number of samples		R^2^		RMSE/%		Accuracy/%	
		Modeling set	Verification set	Modeling set	Verification set	Modeling set	Verification set	Modeling set	Verification set
PLS	4	153	102	0.84	0.81	0.43	0.54	94.7	91.8
PCA	5	153	102	0.57	0.52	0.92	0.98	79.3	75.5
LR	0	153	102	0.49	0.45	1.05	1.23	74.1	69.4

PLS, LR, PCA, R^2^ and RMSE denoted partial least square, linear regression, principal components analysis, determination coefficient and root mean square error, respectively.

**Fig 3:**
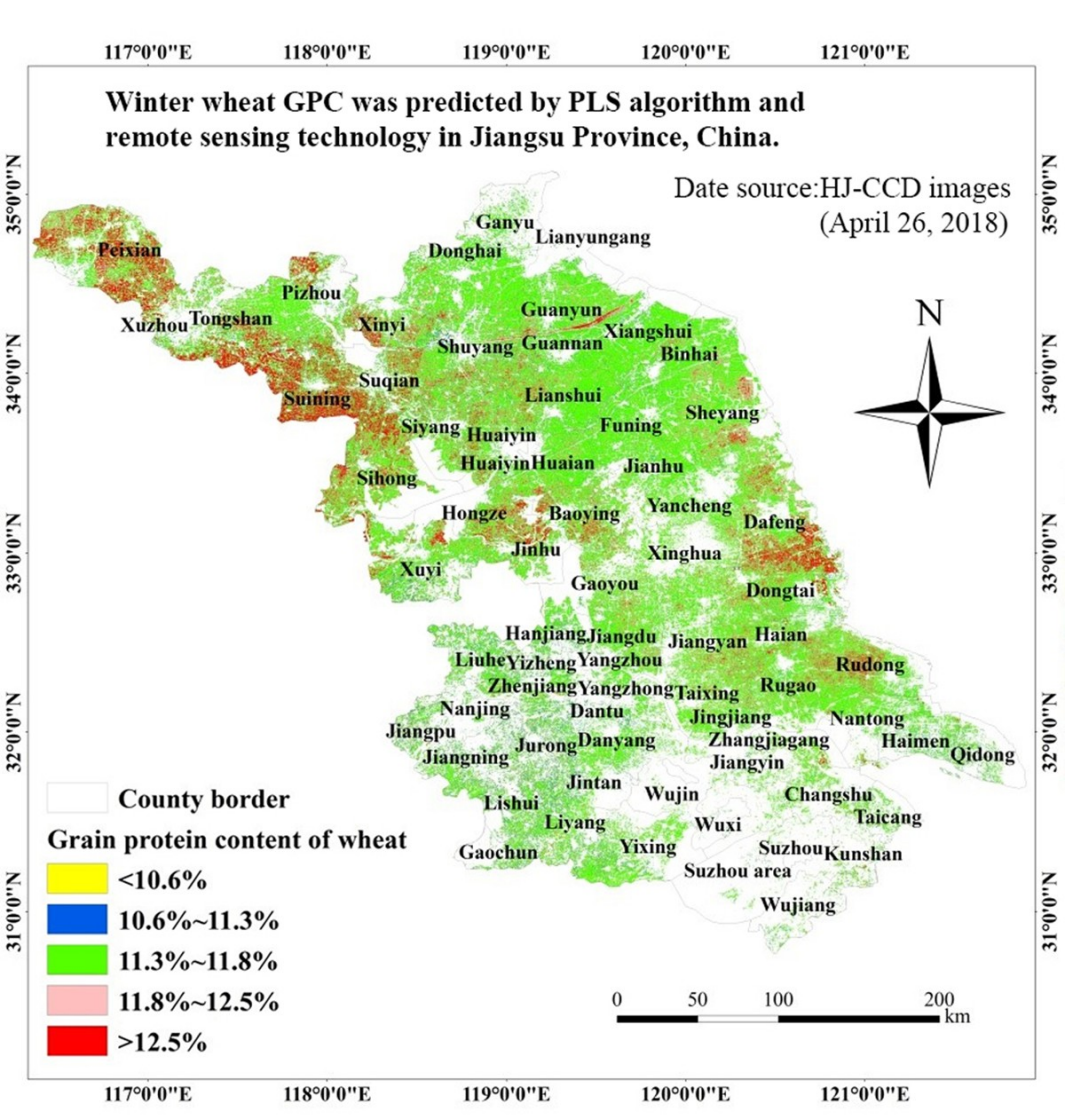
Spatial distribution of winter wheat GPC in Jiangsu province.

According to the above analysis, NDVI, SIPI, PSRI and EVI maps were generated using 2018-04-26 HJ-CCD images. On those the winter wheat planting data was superimposed to remove the non-winter wheat area by one-to-one solution and binarization mask. Based on the administrative boundary vector data, as well as the above PLS model, the spatial distribution map for predicting winter wheat GPC in Jiangsu province was produced (Fig 3). The GPC distribution was mainly 11.3–11.8%. There was often more than 12.5% in Dafeng and Rudong wheat area and the north west of Jinhu wheat area. The GPC of some wheat regions in the north of the Yangtze River was 11.8–12.5%. The number in the southern wheat area was rarely higher than 11.8%. However, the number in the area along the Yangtze River was mainly 11.3–11.8%, particularly in the south.

## Discussion

At present, the remote sensing images used in the crop estimation were mainly originated via MODIS, national oceanic and atmospheric administration (NOAA)/AVHRR, etc. [10, 33, 41]. These images had low spatial resolution and were difficult to apply to high-precision winter wheat remote sensing estimation in small areas. On the other hand, the high-resolution images such as Quickbird (Panchromatic image 0.61–0.72 m, multispectral image 2.44–2.88 m), SPOT (Panchromatic image 10 m, multispectral image 20 m), IKONOS (Panchromatic image 1 m, multispectral image 4 m) were costly [46, 47]. The medium-resolution TM images had revisiting periods of 16 days, making it difficult to obtain high-quality data in time. This limited continuous crop monitoring and made it inappropriate to predict crop quality [38]. The HJ-CCD satellites developed by China have been put into use one after another. The quality of the data obtained was continuously improved and was provided free of charge to users. This has created a convenient data platform for remote sensing and estimation of regional crop’s quality and productivity [8, 48]. The experimental area of the present research has been located in the coastal area along the Yangtze River in Jiangsu Province, China. The whole wheat field has been fragmented and as a result the planting structure was complex. The time resolution of the selected HJ-CCD image was 2 d, and the scanning width of the single scene image was 750 km. These characteristics could meet the estimation demands for the actual regional winter wheat. Considering time resolution, spatial resolution and cost, the HJ-CCD image was more appropriate than the data of MODIS, TM, Quickbird, etc.

There was a close relationship between wheat GPC and 12 remote sensing variables except R_blue_ and R_green_. In addition, there were considerable multiple correlations between all the other remote sensing variables except PSRI and R_blue_, R_green_ and R_red_. This made it difficult to establish a high precision remote sensing estimation model of wheat GPC using traditional algorithms [49]. In this study, the PLS algorithm was used to construct the remote sensing estimation model with NDVI, SIPI, PSRI and EVI as the independent variables. The correlation between the four remote sensing variables and GPC was extremely significant. They could be easily extracted and calculated from the HJ-CCD image. The RMSE values of the GPC prediction model based on NDVI, SIPI, PSRI and EVI as the independent variables were lower than the traditional LR and PCA models. The results showed that the PLS model, as a new multivariate analysis method, has a very high adaptability in wheat GPC prediction. In particular, there were many variables and multiple correlations in the analysis. The PLS algorithm could effectively optimize the dependent variables, and its model was significantly better than LR and PCA algorithms in wheat GPC prediction. The prediction accuracies were above 90%, and were improved by more than 20% compared to the LR algorithm and more than 15% higher than the PCA algorithm. The results were consistent with Hansen *et al*. [19] and Zhao *et al*. [23], and better than Liu *et al*. [21] and Xue *et al*. [22]. In order to reflect it in a better way, the actual situation of field planting and different varieties were selected in the experiment. With the data of different varieties as test samples, the results had more general significance. It was helpful to popularize and apply in practical production.

According to the spatial distribution map for predicting winter wheat GPC in Jiangsu province (Fig 3), the wheat GPC in northern of Jiangsu and eastern of Jiangsu was generally higher. The wheat GPC in the middle area of Jiangsu maintained a medium level of 11.3%-11.8%. The wheat GPC in southern of Jiangsu was relatively low. There was large scale wheat cultivation in northern and eastern of Jiangsu. Local agricultural facilities were well developed, and agricultural production was mainly in the form of farms for planting and management. Therefore, winter wheat planting could be managed uniformly, with good cultivation measures and maximum implementation. Overall agricultural production in the middle area of Jiangsu was slightly worse than that in north of Jiangsu. But the whole structure of agricultural facilities and agricultural production could meet the planting of winter wheat. Therefore, wheat GPC presented a general level range. Southern of Jiangsu was mostly metropolis and urban area with less farmland, and there were few areas for wheat cultivation. At the same time, the local farmland was chaotic and scattered, mainly operated by small farmers households. It might result in good cultivation measures and management could not be used effectively. Therefore, the wheat GPC in southern of Jiangsu was relatively low. The predicted results of the spatial distribution map for predicting winter wheat GPC in Jiangsu province were basically consistent with the actual situation of winter wheat production. It indicated that it was feasible to use the PLS model to predict winter wheat GPC with high precision. It has, therefore, provided an effective method and technical support for the high precision remote sensing prediction of wheat GPC.

The GPC values of the samples used in this study were basically ranging from 10–14%. Samples (GPC) with higher or lower content were relatively few, showing above 14% and less than 10%. There was a lack of samples more than 14.58% and less than 9.36%. If the variation of the GPC samples was increased, the PLS model may be further optimized and its application range would be further expanded. The remote sensing prediction model of winter wheat GPC would become more reliable. The results obtained were based only on the HJ-CCD data of the Jiangsu experimental area. Therefore, whether the model would be applicable to other remote sensing sensor data and/or be able to predict the winter wheat GPC in other areas needs further study.

The present study did not compare the PLS algorithm with artificial neural network (ANN) [50], support vector machines (SVM) [51], geostatistics [52], etc. Simultaneously, it also did not take into account the factors affecting winter wheat cultivation such as weather, soil and cultivation practices and so on. These algorithms and factors might have a wide range of influence on the results of predicting winter wheat GPC and needed further study.

## Conclusion

NDVI, SIPI, PSRI and EVI were sensitive for predicting GPC based on PLS algorithm and HJ-CCD images. Through the modeling set and the verification set evaluation, the GPC model's R^2^ were 0.84 and 0.81 and the RMSE were 0.43% and 0.54%, respectively. The prediction accuracies were above 90%. The improvements were by more than 20% than the LR algorithm and more than 15% higher than the PCA algorithm.

## Supporting information

S1 File Dataset. (XLSX)
